# Enhancing allergenicity risk assessment for novel foods in the EU: insights from the updated EFSA guidance - requirements, knowledge gaps and research needs

**DOI:** 10.3389/ftox.2025.1701391

**Published:** 2025-12-10

**Authors:** Marcello Laganaro, Anna-Maria Pieger, Alejandra Muñoz-González, Estefanía Noriega Fernández, Ermolaos Ververis, Andrea Germini, Antonio Fernández Dumont

**Affiliations:** 1 Novel Foods Team, Nutrition and Food Innovation Unit, European Food Safety Authority, Parma, Italy; 2 Chair of Food Chemistry, Department Chemistry and Pharmacy, Friedrich-Alexander Universität Erlangen-Nürnberg (FAU), Erlangen, Germany; 3 Department of Food and Drug, University of Parma, Parma, Italy; 4 Chief Scientist Office, Executive Director Office, European Food Safety Authority, Parma, Italy; 5 GMO Food Feed Comparative and Environmental Risk Assessment Team, Nutrition and Food Innovation Unit, European Food Safety Authority, Parma, Italy

**Keywords:** novel foods, food allergy, allergenicity assessment, risk assessment, EFSA (European Food Safety Authority), EFSA guidance document, protein safety, food safety

## Abstract

With growing interest in new protein sources, driven by market trends and European Union (EU) food policy priorities, novel proteins are entering the market. These novel foods (NFs) might have the potential of introducing new allergens into the diet. In the EU, the European Food Safety Authority (EFSA) is tasked with assessing the safety of NFs and has recently updated its scientific guidance to reflect advancements in food science and built-up experience in NF risk assessment (RA). EFSA’s allergenicity RA strategy follows a weight-of-evidence approach. Data requirements depend on whether the NF is produced from or with materials containing protein, derived from known allergenic foods (whether subject to mandatory labelling or not), or has unknown allergenic potential. For the latter, a tiered approach focused on assessing the cross-allergenicity potential is applied to single proteins, protein mixtures and whole foods. However, allergenicity RA continues to face challenges including the availability of reference standard materials and the lack of agreement on the interpretation of *in silico* and *in vitro* results, as well as lack of validated and internationally recognized methods meeting regulatory requirements. These limitations underscore the need for further research to deliver robust and fit-for-purpose RA tools to support safe food systems. This review highlights key aspects of the allergenicity RA approach presented in the updated EFSA NF scientific guidance and provides a critical overview of existing uncertainties and emerging approaches aiming at encouraging international cooperation and research to further enhance allergenicity RA.

## 1 Introduction

Innovations in the food sector are increasingly focused on addressing food security challenges and addressing diverse dietary needs. Current market trends and European Union (EU) policy priorities,^1^ ^,^ ^2^ combined with recent scientific and technological advances, demonstrate a growing interest in new protein sources (Aschemann-Witzel et al., 2021; Banach et al., 2023; Siegrist and Hartmann, 2023). However, their introduction into diets may raise allergenicity concerns.

Food allergy is a reproducible immune-mediated adverse health effect triggered by an immune response involving immunoglobulin E (IgE) or non-IgE antibodies following oral exposure to an otherwise harmless antigen, such as dietary proteins (Codex Alimentarius Commission, 1985). It manifests with clinical signs and symptoms of variable severity and duration, negatively impacting the quality of life (Protudjer et al., 2025; Thörnqvist et al., 2019) and, in the worst case, leading to a life-threatening anaphylactic shock. The development of food allergy is preceded by a clinically silent sensitization phase upon a first exposure to the antigen (Valenta et al., 2015) and is discussed to be influenced by several factors, including individual ones (e.g., exercise and alcohol consumption), environmental factors such as allergen exposure and air pollution, and lifestyle factors (e.g., exercise and alcohol consumption) (Munoz-Cano et al., 2020; Pantazi et al., 2023; Peters et al., 2022; Shin, 2021).

In the European population, the point prevalence of sensitization based on specific IgE (sIgE) testing is 16.6%, while food allergy prevalence is 5.7% when assessed by skin prick tests (SPTs) and 0.8% when assessed by oral food challenge (OFC) (Spolidoro et al., 2023). The global impact of food allergies (Warren et al., 2024; FAO and WHO, 2022), alongside the increasing role of protein diversification in the EU food system, emphasizes the need for comprehensive approaches to evaluate the allergenic potential of newly introduced foods and food ingredients. This also supports informed risk-management decision-making, and ensures that food innovation progresses without compromising public health.

According to Regulation (EU) 2015/2283,^3^ foods not significantly consumed in the EU before 15 May 1997, are considered novel foods (NFs). These foods require pre-market authorization and are most often subject to risk assessment (RA) by the European Food Safety Authority (EFSA) to evaluate their safety and any potential nutritional disadvantages under the proposed conditions of use. Allergenicity RA is an integral element of the RA of NFs, as they may introduce new proteins in the diet that could either cross-react with known structurally similar allergens or induce *de novo* sensitization. The latter triggering an allergic response in previously unaffected individuals.

Within the broader framework of food safety, allergenicity RA occupies a distinct position due to the acute and potentially life-threatening consequences of allergic reactions. Unlike other hazards (e.g., microbiological or chemical) which often follow predictable dose-response patterns used for RA, food allergens can provoke severe effects even at very low doses in sensitized individuals, with no consensus threshold of allergological concern currently available for RA (EFSA NDA Panel, 2014; Crevel et al., 2024; Yue et al., 2025). Moreover, compared to other safety endpoints, allergenicity remains disproportionately challenging because immune sensitization mechanisms are complex and still not fully understood (Eiwegger et al., 2019; Renz et al., 2018).

Allergenicity RA currently follows the internationally agreed Codex Alimentarius guidelines (2003–2009) which primarily focus on individual, singularly modified and well-characterized proteins. Since these guidelines were updated over a decade ago, there is a pressing need to revise these methodologies to ensure that safety standards remain relevant and better inform risk management decision-making. Allergenicity RA of NFs is a key area for transdisciplinary research due to complex matrices and protein profiles, with challenges shared globally (Kedar et al., 2024). In the EU, EFSA supports advancement in the field through various actions aimed at ensuring up-to-date, standardized, and comprehensive allergenicity RA.

This review highlights key aspects of the allergenicity RA approach presented in the updated EFSA NF scientific guidance (EFSA NDA Panel, 2024) and provides a critical overview of existing uncertainties and emerging approaches aiming at encouraging international cooperation and research to further enhance allergenicity RA.

## 2 Measures undertaken by EFSA to advance overall allergenicity risk assessment

In recent years, EFSA has made significant investments to advance allergenicity RA. A recent scientific outcome (EFSA GMO Panel, 2022) identified key research needs to support both regulatory and technological developments, thereby informing EFSA’s roadmap for advancing the “weight-of-evidence” (WoE) approach to allergenicity RA. This roadmap marks a substantial conceptual advancement, facilitating the progressive integration of new tools, methodologies, and standardized workflows in allergenicity RA. Ultimately, it aims to enable more robust and comprehensive safety evaluations of regulated products.

Measures undertaken by EFSA to advance allergenicity RA include the publication of different guidance documents (EFSA GMO Panel, 2025; EFSA NDA Panel, 2024) and scientific opinions (EFSA GMO Panel, 2017; EFSA GMO Panel, 2022; EFSA NDA Panel, 2021a) that have helped raise awareness on allergenicity RA. Additionally, several studies procured by EFSA, have contributed to the state of the art (Bebi et al., 2024; Doytchinova et al., 2023; Garciarena et al., 2022; Mackie et al., 2019; Martinez et al., 2024; Mills et al., 2024). These include, for instance, Mills et al. (2024), where a ranking method for clinical relevance and defined screening tools to assess the allergy risk of innovative proteins was proposed.

Moreover, in a recent editorial (Bronzwaer et al., 2025), EFSA outlined key research and innovation needs to advance regulatory scientific areas. With respect to allergenicity RA, these needs include the development of predictive models to support decision-making on cross-reactivity and *de novo* sensitization, and the definition of criteria for when and how *in vitro* and *in vivo* testing should be conducted. These resources are valuable for stakeholders and funding bodies in shaping future research and funding initiatives in the area of food safety. Additionally, EFSA has also stimulated discussions challenging current approaches on allergenicity RA through, e.g., the organization of *ad hoc* sessions at scientific conferences (e.g., IAFP’s European Symposium on Food Safety 2024) or other international events (e.g., OECD Working Party for the Safety of Novel Foods and Feeds, 2025), and the participation in Stakeholder Boards of relevant EU-funded projects.

However, despite EFSA’s actions and the availability of updated strategies, allergenicity RA still often relies on protein-content-driven approaches (Yue et al., 2025).

## 3 Updated EFSA guidance on allergenicity risk assessment for NFs

With regards to NFs, the EFSA Panel on Nutrition Novel Foods and Food Allergens recently adopted the “Guidance on the scientific requirements for an application for authorization of a novel food in the context of Regulation (EU) 2015/2283” (EFSA NDA Panel, 2024). This document outlines data requirements to assess, among others, the allergenic potential of NFs, thereby supporting better informed and, thus, more robust regulatory decision-making processes to protect consumers.

During public consultations of the guidance, EFSA received 715 comments from 47 interested parties, of which 40 comments addressed the allergenicity section,^4^ highlighting public interest in this topic. This updated guidance replaces the superseded versions (EFSA NDA Panel, 2021b; EFSA NDA Panel, 2016) which had a conservative approach to allergenicity RA by assuming that all NFs containing proteins (including peptides) would, by default, have allergenic potential. This approach resulted in allergenicity RA exclusively based on protein content which has been challenged by scientists in the field (Verhoeckx et al., 2016; Verhoeckx et al., 2020; Yue et al., 2025) and food industry (EFSA, 2024) for its limitations.

The updated EFSA NDA Panel (2024) guidance introduces a more pragmatic and evidence-based framework for the allergenicity RA, acknowledging the complexity, heterogeneity and diversity of NFs and the proteins thereof contained. Recognizing the limited understanding of IgE-mediated reactions, the framework employs a WoE approach, incorporating data from scientific literature, bioinformatics, *in vitro* and *in vivo* assays, and clinical studies (Codex Alimentarius Commission, 1985; EFSA GMO Panel, 2010; EFSA GMO Panel, 2011). Data requirements might differ depending on whether the NF is produced from/with materials containing protein, is derived from known allergenic foods (whether or not subject to mandatory labelling according to Regulation (EU) No 1169/2011^5^) or has unknown allergenic potential. For the latter, an evidence-based RA is required, focusing on cross-reactivity, since the potential for *de novo* sensitization remains poorly understood with no validated and/or internationally agreed predictive methods currently available (EFSA GMO Panel, 2017; EFSA GMO Panel, 2022; EFSA NDA Panel, 2024).

**FIGURE 1:**
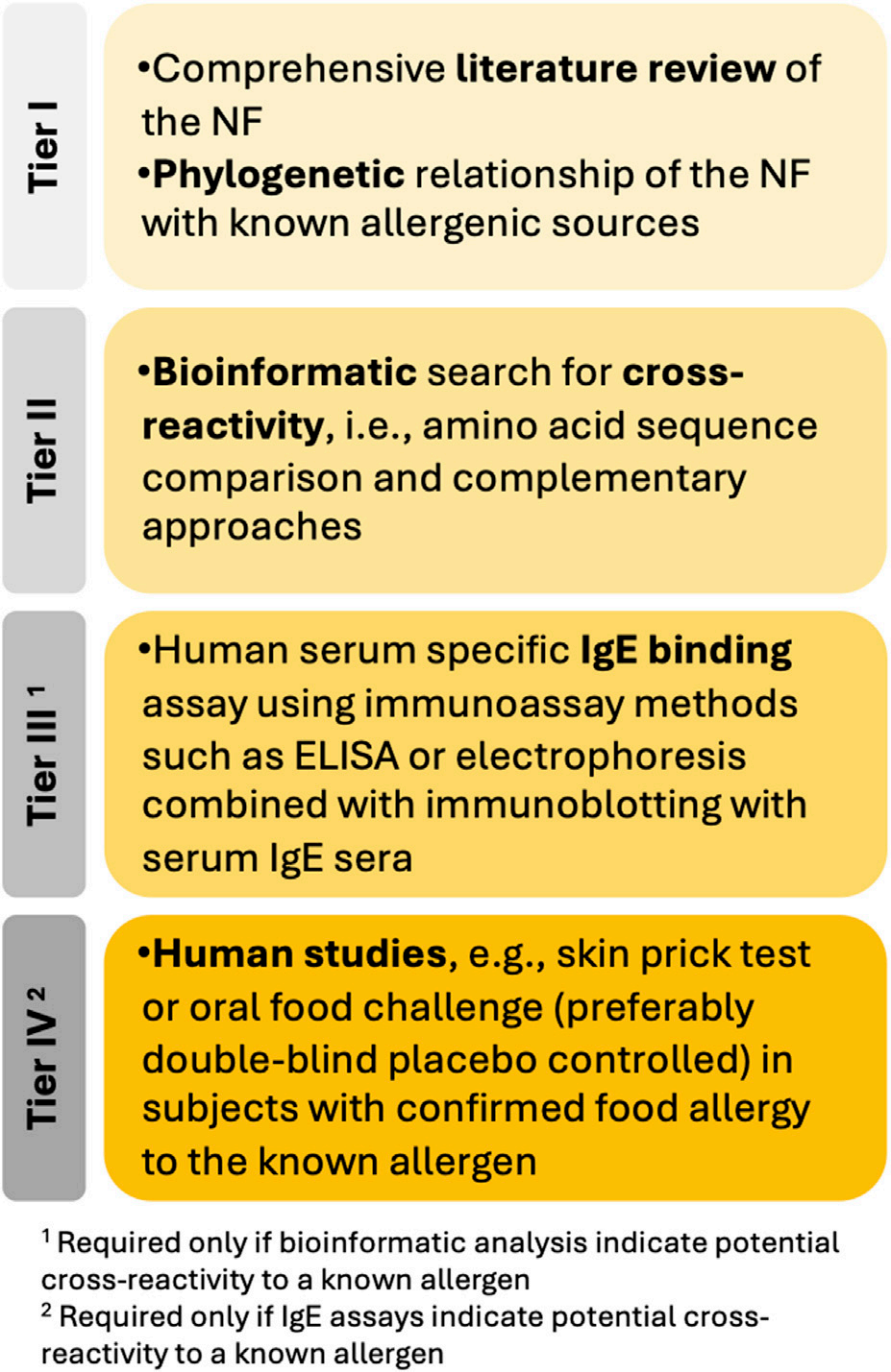
Tiered approach to investigate potential cross-allergenicity of single proteins and simple protein mixtures of unknown allergenic potential (adapted from EFSA NDA Panel, 2024).

The NF matrix and protein composition, particularly in whole foods, introduces additional complexity. Current assessment methods are primarily designed for individual proteins and may be inadequate for complex mixtures or whole foods. To address this aspect, the guidance EFSA NDA Panel (2024) suggests tiered strategies, with different requirements for single proteins and simple mixtures (whose constituents can be fully characterized) (Figure 1), and complex mixtures or whole foods (where not all constituents can be fully chemically characterized and/or identified).

Regardless of the compositional complexity of the NF and its contained proteins, the initial assessment of NF with unknown allergenic potential involves a comprehensive literature review (discussed in the following section). Alongside the literature information, protein digestibility and stability should be considered as a first step to build up an evidence-based evaluation of the allergenic potential of a NF.

The following sections provide a comprehensive overview of all elements to be considered within the WoE framework, as required by the tiered approach outlined in EFSA NDA Panel (2024). This includes considerations about a literature review to inform the allergenicity assessment strategy, about protein digestibility and the tiered approach for NFs with unknown allergenic potential. In the latter, the phylogenetic relationship of the NF with known allergens, *in silico*, *in vitro* and *in vivo* approaches are discussed. Each section explores the rationale behind the need for specific information to support allergenicity RA of NFs, the current tools and methodologies available to generate meaningful data, their limitations, potential for enhancement, alternative approaches, and future directions.

### 3.1 Literature review to inform the allergenicity assessment strategy

The EFSA NDA Panel (2024) requires a comprehensive literature search as a fundamental and first step in the allergenicity RA of NFs containing allergens not subject to mandatory labelling, or those with unknown allergenic potential. In the first case (i.e., known allergens not subject to mandatory labelling), information is required on the prevalence of the allergy, the type and severity of the symptoms, as well as known clinically relevant allergenic proteins of the source and their potency. For NF with unknown allergenic potential, the literature review should encompass the NF, its source, as well as closely related species (as determined by a phylogenetic analysis, explained in Tier I below). All available studies including *in silico, in vitro, in vivo* and human studies (e.g., observational, case reports and/or clinical trials), should be included to gather evidence on their allergenicity. Additionally, different exposure routes, such as skin contact and/or inhalation should be reviewed alongside oral intake.

The aim of the literature review is to provide an overview of existing evidence to a specific question, here, regarding the potential allergenicity of the NF, in a transparent, reproducible, and rigorous way. The search should follow systematic review principles, whenever applicable, and include all pertinent information, as omission of relevant studies may affect the conclusion (EFSA, 2010; EFSA, 2019; EFSA Scientific Committee, 2023; Glanville et al., 2014).

While all available information on the allergenicity of the NF must be evaluated, it is important to note that different study designs contribute differently to the evidence base, with some providing higher-quality and more precise information. In systematic reviews, one critical step is the assessment of the methodological quality of the included studies, typically performed using appraisal tools that identify design or execution features of the study which may introduce bias (EFSA, 2010). Currently, there is no specific appraisal tool for assessing the risk of bias of the different types of studies used to determine the allergenic potential of proteins. Existing resources, such as the ToxRTool^6^ (Schneider et al., 2009) or the Science in Risk Assessment and Policy (SciRAP)^7^ tools that evaluate the reliability of toxicological data from in vivo and in vitro studies, or the OHAT Risk of Bias Tool for human and animal studies^8^, could serve as a guide for the structure and criteria of a future appraisal tool specific for the risk of bias of the available evidence on allergenicity.

**FIGURE 2:**
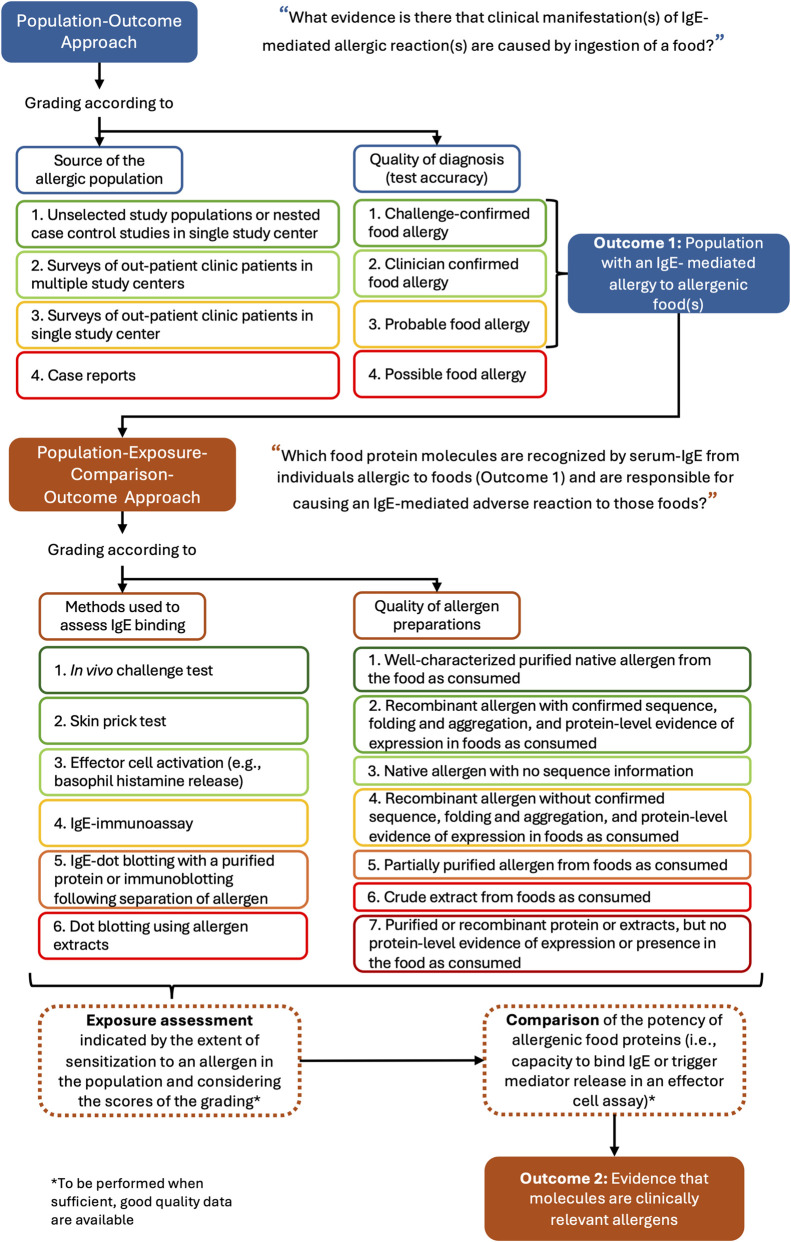
Description of an approach for the grading of allergenicity studies (adapted from Mills et al., 2024). Note: the higher the number given in the grading, the lower the quality of the data for each category.

With the aim of developing novel strategies for predicting the allergenic potential of novel proteins, Mills et al. (2024), carried out a systematic review. For the risk of bias evaluation, studies were assessed simultaneously for the quality of the evidence available and for their clinical relevance, via the introduction of specific criteria. The review followed a two-step procedure consisting of a Population-Outcome and a Population-Exposure-Comparison-Outcome question-formulating framework. As seen in Figure 2, a ranking based on the quality of the diagnosis of the food allergies and on the characteristics of the patient populations used in the studies was carried out for the Population-Outcome approach, while the methods used to assess IgE binding, and the quality of allergen preparations were graded for the Population-Exposure-Comparison-Outcome (Mills et al., 2024).

This type of combined assessment could be of use when evaluating the robustness, reliability and clinical relevance of the evidence available for the allergenicity of a NF, serving as a basis for a fit-for-purpose quality grading tool for allergenicity studies. It must be noted, however, that certain information must be added to make this a comprehensive tool that includes all relevant data and considerations. Among the information to be included is the weight of the route of exposure, the tested item relevance and representativity (e.g., comparable handling to the NF under assessment, phylogenetic proximity to the NF and its source) as well as a clear differentiation, in terms of study requirements, between cases involving known allergens and those involving potential cross-reactivity. Moreover, the inclusion of animal studies, which were excluded from the review from Mills et al. (2024), may be valuable for the development of an all-encompassing appraisal tool.

Carrying out a thorough, non-biased literature review is crucial, not only for obtaining a comprehensive understanding of the available information on the allergenicity of the NF or related sources, but also because it can potentially avoid the need for further testing if sufficiently robust evidence is obtained. The creation and validation of a risk of bias appraisal tool specific to allergenicity studies could minimize unnecessary testing, assist the evaluation of potential allergenic risks associated with NFs, and ultimately reduce costs for the food industry.

### 3.2 Protein digestibility

The role of protein digestibility in allergenicity has been widely discussed. Resistance to acid and enzymatic degradation has been proposed to increase the protein’s potential of triggering an allergic reaction. This has been attributed to the higher probability that resistant proteins reach the cells of the inductive mucosal immune system in their native form or as stable fragments (Bøgh and Madsen, 2016; Foo and Mueller, 2021; Pali-Schöll et al., 2018). However, the relationship between protein digestibility and allergenicity might not be so straight-forward (EFSA GMO Panel, 2021; Remington et al., 2018; Verhoeckx et al., 2019). Factors such as the individual’s age and health status, food processing, matrix composition, and the intrinsic properties of the allergen can be determinant on whether the digestion of the protein eliminates, decreases, has no effect or sometimes even increases its allergenicity (Ballegaard and Bøgh, 2023; Dupont and Mackie, 2025; Rodrigues et al., 2022; Verhoeckx et al., 2019).

The updated NF guidance requires applicants to submit information on protein digestibility to support both the nutritional and allergenicity assessment. The section on nutritional information of the guidance (EFSA GMO Panel, 2024) outlines certain aspects to be considered when selecting an appropriate protein digestibility method. However, overall, standardized *in vitro* protocols to assess the digestibility of foods, capable of producing physiologically relevant, reproducible results, are still lacking. While in the past the pepsin resistance test was widely applied (FAO and WHO Expert Consultation on Allergenicity of Foods Derived from Biotechnology, 2001; FAO and WHO, 2009), it has now been commonly accepted that such a test can only give limited information, and it is recommended to use it in conjunction with *in vitro* digestibility tests that better reflect the conditions inside the gastrointestinal tract (EFSA GMO Panel, 2021; EFSA GMO Panel, 2022; EFSA GMO Panel, 2025).

The INFOGEST static *in vitro* model by Brodkorb et al. (2019) represents a standardized protocol that offers a harmonized approach to study gastrointestinal digestion and covers most of the requirements outlined in the updated NF guidance. It has been proposed as an alternative to *in vivo* models, and a more accessible option compared to dynamic models such as the Simulator of the Human Intestinal Microbial Ecosystem (Rodrigues et al., 2022). However, further refinement is needed, including integration of brush border enzyme activity and characterization of the complex peptide mixtures in the digesta with advanced proteomic tools, such as high-resolution mass spectrometry coupled with sodium dodecyl sulfate–polyacrylamide gel electrophoresis (EFSA GMO Panel, 2017; Martineau-Côté et al., 2024; Mackie et al., 2019). In order to refine the RA of NFs, variables reflecting variations in protein digestibility due to the age of the population of interest could be introduced to improve the gastrointestinal model, as reported in the literature (Ménard et al., 2018; Mackie et al., 2019).

Dynamic systems such as the Tiny-TIM model have also been accepted by EFSA for reporting digestibility of NFs, although mainly for nutritional assessment purpose (EFSA NDA Panel, 2021c; EFSA NDA Panel, 2021d; EFSA NDA Panel, 2021e; EFSA NDA Panel, 2022). This model is a dynamic, multicompartmental system that simulates digestive conditions in the stomach and small intestine that has been shown to provide protein digestibility values that closely resemble those reported for humans (Liu et al., 2024; Santos-Sánchez et al., 2024). Therefore, its potential use in the allergenicity assessment could be considered.

In addition to standardized protocols, there is also a need for a consensus or common understanding on how to interpret the results of these types of tests. A set-back regarding the role of resistance to digestion on allergenicity is that so far, no cut-off value has been defined to establish whether a protein is resistant or not. While some have tried to determine one based on the stability time (i.e., the amount of time it takes for a protein to be undetectable during digestion), large variations on this value have been reported for the same allergens (Bøgh and Madsen, 2016). Therefore, a more precise method is needed for producing reliable results that are of value for RA purposes (EFSA GMO Panel, 2021).

Besides resistance to digestion, the size of the fragments remaining after digestion have been suggested to also play a role in allergenicity. The risk of inducing an adverse allergic response is suggested to increase with the amount of intact protein or large peptide fragments remaining after digestion (Ballegaard and Bøgh, 2023). While peptides must reach a certain size to be allergenic, the exact lower molecular weight limit is not definitively known. However, it is generally accepted that peptides around 3.5 kDa can possess sensitizing and eliciting allergenic potential (Bøgh et al., 2012). The EFSA GMO Panel (2017), recommended that peptides with a size of ≥9 amino acids could be considered for RA, setting a foundation for a minimum eliciting size of peptides. This recommendation is based on the principle that the peptide binding register (i.e., the part of the peptide that binds to major histocompatibility complex (MHC) II) is of 9 amino acids, and therefore peptides shorter than this are unlikely to cause an allergenic reaction (EFSA GMO Panel, 2017). Nevertheless, evidence shows that even smaller fragments can sometimes elicit reactions, prompting a conservative approach in assessing NFs to include as many potential allergens as possible (Bøgh et al., 2012; EFSA GMO Panel, 2017; Mackie et al., 2019; Remington et al., 2018; Verhoeckx et al., 2019).

Aggregation propensity is also important for assessing the safety of small peptides, including their immunogenicity (De Groot et al., 2023). This is the case also for proteins that are easily digestible. In fact, they could retain the sensitizing potential through peptide aggregation in the digestive tract, which can mimic the intact molecule or create a synergistic effect when most peptides of the digests are present together (Bøgh et al., 2012). Therefore, investigating and evaluating the aggregation potential of peptides and/or the sensitization capacity of potential aggregates present in the digesta is essential to have a comprehensive RA of NFs of proteinaceous nature within the body of evidence context.

The abundance of allergens can also influence the allergenic potential of a food. Abundance may be interpreted in one of two ways: as the amount of allergen originally present in the source food or as the concentration of allergenic peptides in the digesta. In either case, abundance is closely related to exposure. The likelihood of exposure to intact or fragmented proteins varies with the stage of digestion. Exposure to intact allergenic proteins is more likely to occur during the oral phase, while gastrointestinal exposure may involve intact proteins that are resistant to digestion or allergenic fragments resulting from it (Verhoeckx et al., 2019). While some studies have found a linear correlation between the level of exposure and sensitization, exceptions exist where a dose-response relationship could not be established. Several factors, such as the biophysical properties of the allergen, the route and mechanism of sensitization, and the frequency and duration of exposure can affect this relationship. Therefore, the presence and concentration of peptides in a food, even after its digestion, can only be viewed as a contributing factor rather than a definitive predictor of allergenic potential (Fernandez et al., 2019; Foo and Mueller, 2021; Wang et al., 2021).

**FIGURE 3:**
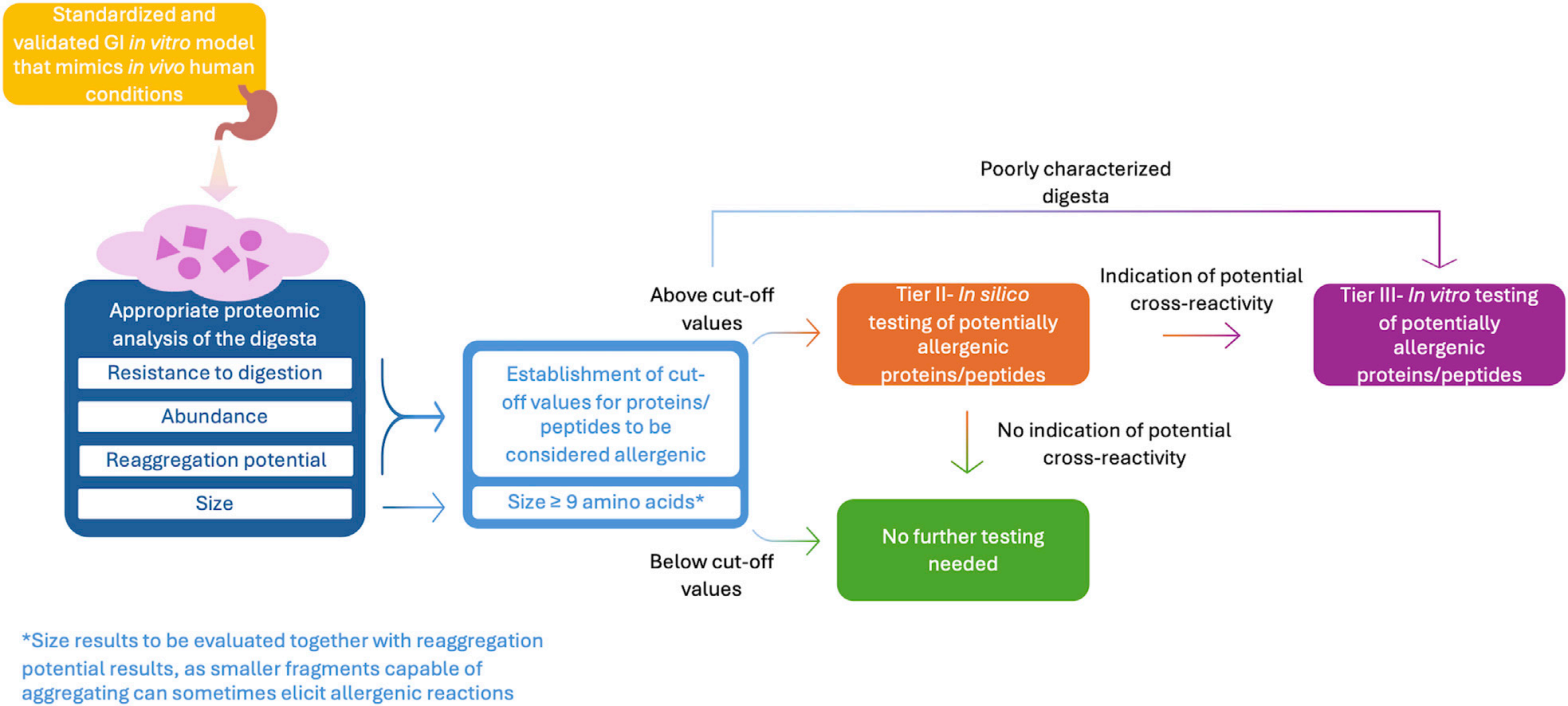
Proposed approach for the assessment of protein digestibility within the allergenicity RA.

While there is a general agreement that resistance to acid and enzymatic degradation during digestion is somehow a factor to be considered when assessing a protein’s allergenic potential, more research is needed to determine the real extent and direction of this relationship. Continued advances in physiologically relevant *in vitro* digestion models, as well as in the interpretation of their results such as the establishment of cut-off values to determine the size, abundance and stability of proteins considered allergenic/adverse, are essential. Once these cut-off values are determined, an approach like the one proposed in Figure 3 could be followed during the allergenicity RA.

It must also be noted that assessing protein stability during digestion alone is insufficient to predict allergenicity. As suggested in Figure 3, these methods should be followed by either *in silico* testing or immunological assays conducted on the digesta (see Tier II–*in silico* approaches and Tier III–*in vitro* testing) to determine, not only the presence of stable proteins and peptides, but also their sensitizing and eliciting capacity. The sequential immunological testing of the digesta and/or refined portion of it is crucial to evaluate the clinical relevance of the proteome left after digestion. Together, these developments are key to better understanding how different factors may influence protein digestibility and how this, in turn, may affect their immunogenicity and protein safety. Artificial intelligence (AI) will most likely play a key role in the development of such future models by facilitating the identification of relationships between results from analytical techniques used to characterize the digesta and the probability of allergenicity of the resulting proteome (Bøgh and Madsen, 2016; Mackie et al., 2019). However, while the application of AI in the field of allergenicity RA is promising and advances are being made in the area, data scarcity is still a limiting factor for their application. For AI systems and models to be trustworthy, they need to be built upon large quantities of high-quality and representative data, to avoid inaccurate and/or biased predictions (van Breugel et al., 2023).

### 3.3 Tiered approach for novel foods with unknown allergenic potential

#### 3.3.1 Tier I - phylogenetic relationship to known allergens

Information on phylogenetic relationships between the NF and other known allergens is requested as a first step on the allergenicity RA of NFs with unknown allergenic potential. Evolutionary conserved protein structures and functions suggest similar bioactivity among phylogenetic groups, showing that possible adverse health effects can be associated to phylogeny (EFSA GMO Panel, 2025; Mills et al., 2024). Understanding the phylogenetic origin of a novel protein can direct a focused testing strategy, aiming at elucidating whether a protein can present an allergenic risk to consumers sensitized and/or allergic to phylogenetically related foods.

To investigate the phylogeny of proteins, molecular and morphological methods can be applied. In order to have a full evolutionary picture, both phenomic or genomic data should be considered together (Keating et al., 2023). Particularly, the use of DNA sequencing has been proven to enhance correlations between IgE sensitization and phylogenetic proximity when compared to more traditional phenomic-based approaches (Levetin et al., 2016). Studies have also confirmed cross-reactivity across various subphyla (Pali-Schöll et al., 2019; Verhoeckx et al., 2014). Recent research by Letovsky et al. (2024) indicates that while phylogenetic distance can account for co-sensitization among species, there are notable exceptions. A close phylogenetic proximity does not always align with a shared immune response (Seidler et al., 2024), and conversely, phylogenetic distance does not preclude common immune reactions. Homologous proteins with specific structural features capable of inducing cross-reactivity may be present in unrelated sources, such as the case of birch pollen-allergic individuals reacting to proteins from the *Rosaceae* family (Kondo and Urisu, 2009; Muluk and Cingi, 2018). Therefore, combining phylogenetic analysis with structural similarity assessments could improve allergenicity risk evaluations (Chapman et al., 2007; Lorenz et al., 2009).

Particularly for microorganisms and microbial proteins, phylogenetic considerations alone provide limited predictive power for allergenicity due to the under-characterization of microbial allergens. Existing databases primarily focus on plant and animal allergens, leading to restricted phylogenetic inferences for microbial proteins due to a lack of reference data. An analysis conducted on the five major allergen databases (WHO/IUIS, AllFam^9^, AllergenOnline,^10^ SDAP,^11^ and AllerBase^12^) confirmed that microbial allergens are poorly represented across all platforms, constraining the predictive power of phylogenetic allergenicity assessments for microbial proteins (Sarkar et al., 2023). Moreover, horizontal gene transfer can introduce, by exchange of genetic material between organisms, traits such as genes encoding biogenic amines, which are known to cause food intolerance/allergy (Rossi et al., 2014). The acquisition of such metabolic pathways via horizontal gene transfer underscores the potential for microbial proteins to carry allergenic/immunogenic properties in otherwise innocuous species that may pose health risks. This behaviour further complicates allergenicity predictions via phylogenetic analysis and highlights the need for more research to understand the reliability of phylogenetic assessments for allergenicity purposes in microorganism-derived NF.

Overall, while a close phylogenetic relationship cannot definitively prove cross-reactivity, principally for plant and animal-derived NFs and proteins, it serves as a valuable indicator and supports the need for further testing.

#### 3.3.2 Tier II - *in silico* approaches

*In silico* methods are valuable for evaluating potential cross-reactivity of proteins, helping to decide whether experimental validation of their clinical relevance is necessary. Molecular allergology allows the detection of individual patterns of sensitization. Bioinformatic analyses, which involve comparing amino acid sequences of novel query proteins against known allergens using computational tools and databases, are required for NF composed of single proteins and simple mixtures with unknown allergenicity potential (EFSA NDA Panel, 2024).

According to EFSA requirements and Codex Guidelines, a sequence identity of at least 35% over 80 amino acids rises concern for potential cross-allergenicity requiring further investigations (FAO and WHO, 2009; EFSA GMO Panel, 2010; EFSA GMO Panel, 2011; EFSA NDA Panel, 2024). However, studies indicated that cross-reactivity usually occurs with more than 70% sequence identity to a known allergen, but it can also occur below 35%, which might result in false positive or negative results (Aalberse, 2000; Goodman, 2006; Dubiela et al., 2018).

Beyond Codex Alimentarius guidelines, various approaches for predicting cross-reactivity have been proposed in the literature. Several reviews have summarized the tools and databases available for this purpose (Bucholska et al., 2018; Hayes et al., 2015; Radauer, 2017; Radauer and Breiteneder, 2019). These include alignment-free methods based on the main physicochemical properties of proteins (Dimitrov et al., 2013 - AllergenFP^13^) (Dimitrov et al., 2014 - AllerTOP^14^), detection based on filtered length-adjusted allergen peptides (Soeria-Atmadja et al., 2006; Martinez Barrio et al., 2007 – ELLAVER^15^), pairwise sequence similarity vectorization (Muh et al., 2009 - AllerHunter^16^), and amino acid/dipeptide composition of proteins for IgE epitope mapping (Saha and Raghava, 2006 - AlgPred^17^). Some allergen databases specifically developed to assess NF proteins for potential cross-reactivity have also been developed (Goodman et al., 2016 – AllergenOnline^18^). Additionally, quantitative structure–activity relationship models have been applied to predict linear protein epitopes and their IgE binding potential (Yu et al., 2023). Quantitative structure–activity relationship models may be particularly interesting due to their established use in regulatory science for predicting (eco)toxicological endpoints based on structural similarities. However, there is currently no evidence that alternative models and tools provide more accurate or reliable results than traditional FASTA sequence comparison using a comprehensive allergen database with the criteria of >35% identity over 80 amino acids (Mazzucchelli et al., 2018).

Current limitations in databases and tools for *in silico* allergenicity prediction of novel proteins are well-documented (Fernandez et al., 2021; EFSA GMO Panel, 2022; EFSA GMO Panel, 2025). A significant challenge is the absence of a standardized search engine or methodology, leading to inconsistencies across different bioinformatic approaches. Differences in objectives, data types, accessibility, annotation levels, and applications hinder the comparability of databases. The input data used are heterogeneous, originating from various sources, complicating integration and standardization (Mazzucchelli et al., 2018; Verhoeckx et al., 2020; Remington et al., 2018; Ivanciuc et al., 2011; Fernandez et al., 2021; Radauer and Breiteneder, 2019).

*In silico* hits do not always correlate with clinically relevant outcomes, underscoring the need for a curated database that ranks allergen sequences by clinical relevance (Fernandez et al., 2021). A recent systematic review described by Mills et al. (2024) resulted in a curated set of allergens ranked by clinical relevance, which is crucial for future refinement of bioinformatic tools. Although challenging to develop, integrating these findings into a curated, harmonized database ranking allergens by clinical relevance is necessary to streamline bioinformatic approaches, ensuring faster, more reliable and more transparent allergenicity RA (Fernandez et al., 2021; EFSA GMO Panel, 2022; Mills et al., 2024; EFSA GMO Panel, 2025). Attempts were made with the WHO/IUIS Allergen Nomenclature^19^ where clear parameters were outlined for obtaining the official designation of a new allergen (Goodman and Breiteneder, 2019).

Uncertainties remain in evaluating the allergenic potential of novel proteins, as the structural features responsible for clinically relevant reactions are debated, and the mechanisms underlying immune responses are not fully understood (Crevel et al., 2024; Turner et al., 2022). Thus, this also results in the lack of defined structural characteristics to distinguish allergens from non-allergens, leading to model validation inconsistencies due to varied control protein sets (Fernandez et al., 2024; Remington et al., 2018). Additionally, only a few databases are continuously updated to incorporate recent discoveries, while most are curated occasionally, reducing the reliability of the bioinformatics searches (Hayes et al., 2015; Mazzucchelli et al., 2018; Remington et al., 2018; Verhoeckx et al., 2020). Attempts in the developments of databases, which aimed at being constantly up to date are, e.g., COMPARE^20^ updated yearly (van Ree et al., 2021), AllergenOnline,^21^ and Allergome^22^ which offers the real time monitoring of IgE sensitization module (Mari et al., 2006). Allergen databases have also been proven to be particularly deficient in microbial allergens’ characterization hindering even further the predictive power of sequence-based allergenicity assessments for microbial-derived proteins and NFs (Sarkar et al., 2023). Overall, the databases are inconsistent with each other leading to variable and, sometimes, contradictory results depending on the database used; certain areas, such as microbial allergens, are not fully covered.

Protein structure prediction remains a complex, interdisciplinary topic of interest. Most current approaches focus on linear epitopes and do not assess potential conformational ones. However, understanding the 3D structure of an allergen can reveal the location of epitopes and their potential interactions with IgE antibodies. This information can significantly impact the prediction of allergenicity (Saetang et al., 2022; Varadi et al., 2022). Recent advancements in deep learning have improved prediction accuracy through software tools like AlphaFold (Senior et al., 2020) and AplhaFold2 (Jumper et al., 2021). The updated NF guidance additionally mentions alternative methods, such as 3D structure analysis and machine learning for conformational IgE epitope mapping and matching (EFSA NDA Panel, 2024; EFSA GMO Panel, 2022). However, further research is still needed to enhance knowledge on protein folding prediction before its applicability in regulatory science practices such as RA of NFs (Huang et al., 2023).

Finally, current *in silico* practices are approaches typically designed for assessing a few newly expressed proteins introduced by conventional transgenesis. This presents challenges for NFs often consisting of partly uncharacterized complex protein mixtures (Moreno et al., 2024; Verhoeckx et al., 2016; EFSA GMO Panel, 2025). To mitigate these limitations, databases are increasingly incorporating protein entries derived from proteomic and genomic analyses, enhancing the ability to characterize the proteome of NFs *in silico*. Despite these advancements, the inclusion of such data remains limited, posing challenges for their effective use in risk assessment. Yet, the use of untargeted proteomic approaches linked to predictive algorithms has been proven to serve the purpose of characterizing the protein fraction comprehensively and have already been applied in the allergenicity assessment context (Bianco et al., 2022; D’Auria et al., 2023; EFSA GMO Panel, 2022; Fernandez et al., 2021; Mazzucchelli et al., 2018; Nedyalkova et al., 2023; Verhoeckx et al., 2020). However, to obtain reliable results, a robust and efficient method for protein classification is needed, highlighting once again the long-standing the need for a centralized reference allergen database (Brusic et al., 2003).

In summary, the successful application of *in silico* tools for predicting cross-allergenicity in the RA of NFs relies heavily on access to updated, curated, and harmonized databases. These databases should ideally prioritize allergens based on their clinical importance, recommending further testing when *in silico* hits are identified, according to their ranking. Despite advancements in proteomic analysis and AI, the efficient storage and analysis of data represent significant challenges in allergen bioinformatics (van Breugel et al., 2023; Radauer and Breiteneder, 2019). These challenges are particularly evident when evaluating the cross-reactive potential of NFs with unknown protein components and microbial-derived NFs. Nonetheless, once robust and comprehensive proteomic and genomic analytical data are integrated into *in silico* databases, their application in the allergenicity risk assessment could be highly valuable for characterizing and assessing NFs allergenicity potential. Thus, developing specialized databases and advanced predictive algorithms is crucial for improving the accuracy of bioinformatic tools and enhancing our understanding of the molecular basis of cross-reactivity (van Breugel et al., 2023). Attempts in developing computational models for prediction of novel proteins’ *de novo* sensitizing potential have also been made (Li et al., 2023). However, whether these developments will also aid clarifying why certain proteins become allergens and help predicting their clinical relevance is still uncertain due to the early stage of research. Collaboration within the scientific community to harmonize data standards, ensure regular updates, and validate predictive algorithms is crucial for advancing bioinformatic allergenicity assessments approaches for NFs and novel proteins.

#### 3.3.3 Tier III - *in vitro* testing

According to the updated guidance, in case bioinformatic searches suggest potential cross-reactivity, follow-up IgE binding analyses using human serum should be conducted with the NF (EFSA NDA Panel, 2024).

**FIGURE 4:**
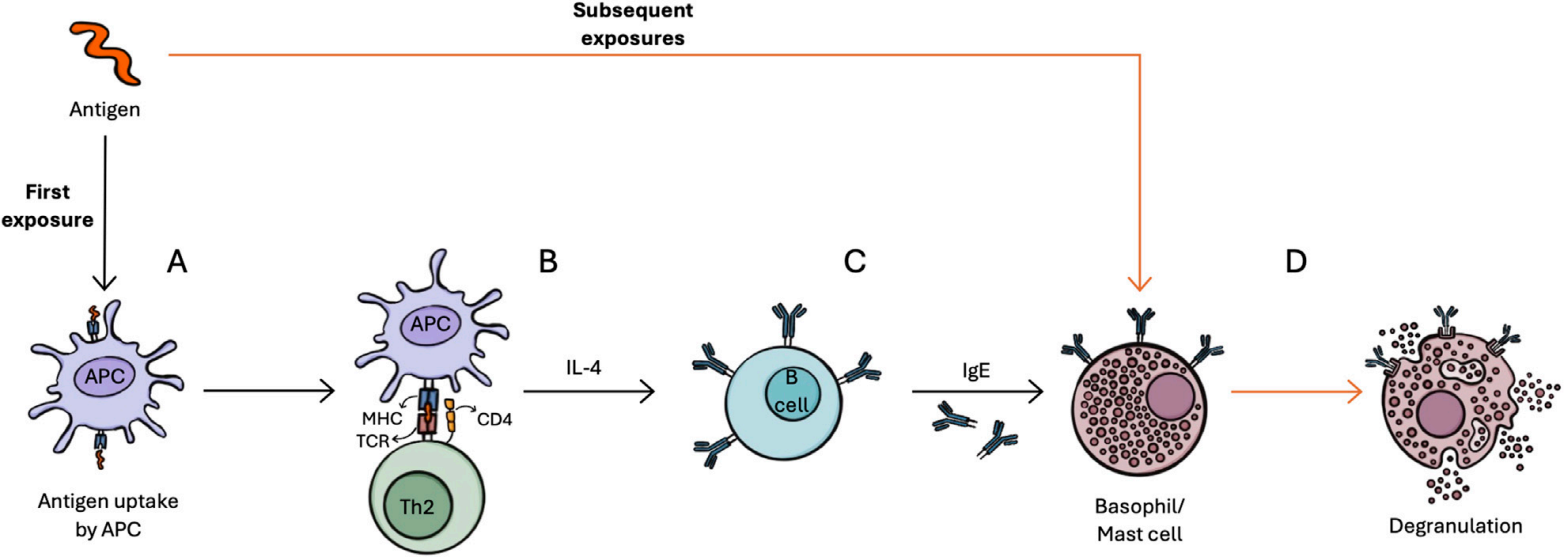
Stages of IgE-mediated allergy development after exposure to an antigen. Upon first exposure, antigens are processed by antigen presenting cells (APCs) **(A)**. APCs present the antigen via their major histocompatibility complex (MHC) to T cells, which recognize the antigen/MHC-complex via the T cell receptor (TCR). This binding is reinforced by cluster of differentiation (CD)4 and CD8 molecules (here shown CD4) **(B)**. Specialized subsets of T cells (e.g., T helper cells 2 (Th2)) release cytokines and interleukins (e.g., IL-4), which stimulates B cells to differentiate and produce specific IgE antibodies **(C)**. Subsequent exposure to the antigen leads to cross-linking of IgE antibodies on basophils and mast cells, which leads to degranulation and the release of immune mediators **(D)**.

IgE-mediated allergy development occurs in two phases as shown in Figure 4. First, exposure to the antigen triggers a clinically silent sensitization phase, involving the production of specialized memory T cell subsets such as T helper cells 2 (Th2) (Figure 4A). Cytokines and interleukins (e.g., IL-4) released by these cells stimulate B cells to produce specific IgE antibodies, which bind to receptors on mast cells and basophils (Figure 4B). Subsequent exposure to the antigen cross-links IgE antibodies (Figure 4C), resulting in degranulation of mast cells and basophils, releasing immune mediators such as histamine (Figure 4D) (Renz et al., 2018; Saidova et al., 2018; Valenta et al., 2015; Zhou et al., 2021).

To assess the levels of sIgE in allergic individuals, different assays can be employed, including enzyme-linked immunosorbent assay (ELISA), radioallergosorbent test, and immunoblotting (Verhoeckx et al., 2016). These assays typically involve a solid phase where the allergen extract or recombinant allergen is immobilized, followed by the addition of IgE from the patient’s serum (produced during Figure 4C phase), anti-IgE antibodies, a label for the anti-IgE antibodies, a substrate to produce a measurable signal, and a relevant signal-reading system (Arsenis et al., 2025; Mazzucchelli et al., 2018). Suitable methods for NF RA purposes listed in the guidance include ELISA or electrophoresis with immunoblotting (EFSA NDA Panel, 2024).

To evaluate potential cross-reactivity previously identified via phylogenetic relationship and/or bioinformatic approaches, sera from individuals with clinically verified allergies to the identified related foods can be used (e.g., serum from shrimp-allergic patients when testing insects). Additionally, a negative control group with sera from non-phylogenetically related allergies (e.g., peanut, when testing insects) should be included to exclude non-specific IgE binding (Verhoeckx et al., 2016). Although these assays are to date among the most commonly used *in vitro* tools to investigate the IgE-binding capacity of proteins (Riggioni et al., 2024), clear guidelines on conducting these tests, including aspects such as obtaining sera for rare allergens, using individual or pooled sera, selecting the test population, and utilizing negative controls to eliminate non-specific IgE binding, are still needed (EFSA GMO Panel, 2022; Moreno et al., 2024; Remington et al., 2018; Verhoeckx et al., 2016). When assessing potential cross-reactivity, careful consideration should be given to the selection of subjects, serum, and establishment of suitable positive and negative controls. Serum from well-characterized clinically relevant allergic patients is necessary to identify potential allergens (Verhoeckx et al., 2016). A proportion of subjects may be sensitized but not clinically allergic. Using sensitization as the hazard metric may lead to overly conservative and protective assessments, whereas choosing the development of allergy as the endpoint may provide a more accurate representation (Remington et al., 2018). Furthermore, subjects can be allergic to multiple foods, exhibiting different sIgE titers, recognition patterns of specific allergens, and varying degrees of allergy severity (Remington et al., 2018). Another general limitation of sIgE assays is the potential interference from substances such as hemoglobin, lipids, and bilirubin. Additionally, false-negative sIgE results can occur in patients with high concentrations of circulating biotin (Vitamin B7) due to regular supplementation (Arsenis et al., 2025).

As mentioned earlier, the presence of IgE antibodies is a prerequisite for allergy development, but IgE binding alone does not necessarily indicate clinically relevant reactions (Arsenis et al., 2025; Verhoeckx et al., 2016). Therefore, IgE binding assays alone cannot rule out or confirm a food allergy as they cannot distinguish between sensitized and allergic individual (Anvari et al., 2019; Renz et al., 2018). Thus, follow up *in vivo* methods to IgE-binding assays are needed to correctly interpret the results and to make a definitive statement about the allergenic potential of a NF.

##### 3.3.3.1 Emerging *in vitro* tests

While the updated NF guidance mentions only ELISA and immunoblotting for Tier III testing, it is important to acknowledge that other *in vitro* tests are available. Even though these assays still require further validation, they may prove useful in the future for assessing the allergenicity of NFs.

The basophil activation test (BAT) can be used as an alternative or complementary tool to IgE-binding tests (Santos et al., 2023; Verhoeckx et al., 2016). The BAT involves assessing the proportion of activated basophils in the patient’s blood by analyzing primary activation markers. Basophils are activated through the cross-linking of IgE antibodies by antigens, triggering degranulation and subsequent fusion of vesicles with the cell membrane, leading to the release of immune mediators such as histamine (Figure 4D). This process results in distinct phenotypic changes, which can be assessed via flow-cytometry (Santos and Shreffler, 2017; Santos et al., 2021). This functional assay provides a more precise measure of allergic reactivity than quantifying sIgE concentrations (Santos et al., 2021). Notably, the BAT can distinguish between sensitized yet tolerant individuals and those with allergic reactions, making it a valuable tool to support the diagnosis of food allergies (Santos et al., 2014). Drawbacks of the method include the need that the assay must be performed within 24 h of blood collection, ideally 3–4 weeks after an allergic reaction occurred. Furthermore, about 10% of allergic individuals are classified as non-responders making the results uninterpretable (Mayorga et al., 2024; Santos and Shreffler, 2017). Currently, the BAT is mostly used as a research tool with a lack of standardized protocols, including identification and activation markers, allergen sources and used concentrations (Ebo et al., 2024). This provides an additional challenge in interpreting results and comparing between studies (Ebo et al., 2024), which is hindering its applicability in RA procedures. Although BAT results may be considered and further substantiate the WoE regarding the allergenicity potential of a NF, further validation from multicentric studies with a large sample size and well-characterized donors, as well as standardized laboratory practices and flow-cytometry data analysis is required before the readout of this assay can be used unequivocally for regulatory science purposes (Arsenis et al., 2025; Santos and Shreffler, 2017).

Another emerging alternative to IgE-binding assays is the mast cell activation test (MAT). MAT differs to BAT for the starting cell population. Instead of basophils, mast cells (MCs) from cell lines or primary MCs derived from healthy donor peripheral blood progenitors are used. MCs are also IgE effector cells, but they are more challenging to access due to their tissue-resident nature (Arsenis et al., 2025). Upon activation of MCs with allergens (Figure 4C), activation markers are measured via flow cytometry (Arsenis et al., 2025). In comparison to the BAT, MAT offers the advantage of not showing non-responders, leading to conclusive results in all individuals tested (Ebo et al., 2024; Santos et al., 2018). On the other hand, MAT requires lengthy cell culture (2–4 weeks for cell lines, 4–10 weeks for primary cells) (Arsenis et al., 2025), which requires costly suitable laboratory facilities (Ebo et al., 2024). In studies analyzing peanut allergy, BAT and MAT showed a higher accuracy in diagnosing food allergy compared to sIgE testing (Ebo et al., 2024). Comparison between BAT and MAT regarding sensitivity is leading to ambiguous results (Bahri et al., 2018; Santos et al., 2018), proving that further validation might be needed. Until now, research on the utility of MAT is limited, with most studies focusing on peanut allergy and perioperative hypersensitivity reactions (Ebo et al., 2024). Due to these limitations, further investigation is still needed to fully explore the potential of MAT (Arsenis et al., 2025; Santos et al., 2023), including standardization in the different source materials used, activation makers, laboratory workflows, test material and reporting and interpretation of data (Ebo et al., 2024).

T cells play a pivotal role in mediating specific immune responses and forming immunological memory. Understanding their frequencies, phenotypes, and functional capacities is essential for assessing an individual’s immune status in the context of food allergy. Upon encountering an antigen, on T cell receptor (TCR) sites, T cells recognize short peptide antigens presented by antigen-presenting cells (APCs) via their MHC (Figure 4A). The binding of the MHC-TCR complex is reinforced by cluster of differentiation (CD)4 or CD8 molecules. CD4^+^ T cells, differentiate into various subclasses, including Th2 cells, upon activation (Jutel and Akdis, 2011; Ruiter and Shreffler, 2012; Kennedy, 2010; Zhou et al., 2021; Saidova et al., 2018). Th2 cells are mainly associated with the development of food allergies (Saidova et al., 2018). Detecting antigen-specific T cells, however, poses a challenge due to their low frequency in the periphery of food-allergic individuals (<20/million CD4^+^ T cells) (Bacher and Scheffold, 2013; Saidova et al., 2018). To overcome this, highly specific labelling methods and sufficient input cell numbers are required. Several approaches can be applied. Up to date, MHC multimer staining is the current gold standard for identifying antigen-specific T cells, particularly suited for CD8^+^ T cells, less suited for CD4^+^ T cells, which are discussed to be mainly involved in the development of food allergies (Poloni et al., 2023). A major drawback of this testing approach is that a defined peptide is restricted to a particular MHC haplotype, resulting in the necessity that the antigenic epitope must be known and characterized in detail (Archila et al., 2016; Bacher and Scheffold, 2013). Particularly in the case of NFs being often constituted by complex mixtures, this can be difficult to achieve. Activation-induced marker (AIM) assays offer an alternative approach, which involves measuring the expression of activation markers on the surface of T cells via flow-cytometry after *in vitro* stimulation of peripheral blood mononuclear cells (Aparicio-Soto et al., 2020). This method does not require prior knowledge of allergenic epitopes or their MHC restriction, allowing the use of single peptides, proteins, or whole antigen lysates. The AIM assay is particularly useful for analyzing T cell responses against complex antigens, which contain multiple T cell epitopes (Frentsch et al., 2005; Poloni et al., 2023; Bacher and Scheffold, 2013). Cytokine staining is another approach, which measures cytokine production by T cells upon activation. Although, cytokine production may be restricted to certain T cell subsets, and not all specific T cells produce the cytokines of interest. Therefore, relying solely on cytokine production to enumerate antigen-specific T cell frequencies may be incomplete and requires careful interpretation (Bacher and Scheffold, 2013; Poloni et al., 2023).

In summary, understanding the role of T cells in food allergy is crucial, and detecting antigen-specific T cells requires highly specific labelling methods. While MHC multimer staining is the gold standard, cytokine staining and AIM assays offer alternative approaches, each with its strengths and limitations.

Overall, *in vitro* studies are a less invasive, and more cost-effective alternative to provocation studies. Compared to established IgE-binding assays, emerging IgE-tests like the BAT and MAT offer the advantage that they can distinguish between sensitization and allergy *in vitro*, although further validation is needed in both assays (Bahri et al., 2018; Santos et al., 2014; Santos et al., 2018). Additionally, compared to provocation tests like the SPT (described in section Tier IV–*in vivo* studies), different protein extracts and stringent buffers can be used (Verhoeckx et al., 2016), and specific protein fractions of interest can be tested, after applying appropriate separation techniques to the NF sample. A comprehensive understanding of cellular immune responses is crucial for accurately assessing IgE-mediated food allergy. T cell-based assays could offer, in the future, new opportunities in assessing different end points of allergic reactions and broadening the understanding of the immune system. Emerging *in vitro* tests such as the BAT and MAT, as compared to conventional IgE-binding assays, may offer the advantage to make considerations on the clinical relevance of allergic reactions. Thus, on a long term, ideally, such functional assays may have the potential to reduce or even substitute time-consuming and resource-intensive *in vivo* testing in the evaluation of potential allergenic risks associated with NFs. Overall, although offering opportunities and further support the WoE regarding the allergenicity potential of a NF, each *in vitro* method lacks standardization and validation in the laboratory practice, as well as in agreement in the readout of results before they can be implemented in the RA process.

#### 3.3.4 Tier IV - *in vivo* studies

According to the NF guidance, a positive result according to the *in vitro* assays mentioned in Tier III, should be followed up by *in vivo* human testing. Provocation tests such as a SPT or an OFC should be conducted in individuals allergic to foods that contain proteins which share a high amino acid sequence homology to the NFs proteins (identified in Tier II) that tested positive *in vitro* (identified in Tier III).

SPTs allow screening individuals for the presence of food-specific IgE antibodies. Allergen extracts or fresh food are applied to the individual’s skin and the resulting wheal size is measured, which reflects the amount of mast cell mediators released in response to the allergen (Santos et al., 2023; van der Valk et al., 2015; Renz et al., 2018). SPT is commonly used in supporting food allergy diagnosis due to its simplicity, low cost, and rapid readout (Appel et al., 2018). The reliability of SPT in identifying food allergies depends on the specific allergen being tested. For example, SPT is well-suited for assessing peanut and cashew nut allergies. In contrast, it has limited accuracy for other foods, such as wheat and soy, due to the scarcity of data and variability in test performance (Santos et al., 2023). Using fresh food for SPT can increase sensitivity, making it a valuable diagnostic tool (Santos et al., 2023). Yet, the allergens or extract materials used and the methods for performing and recording SPT are not standardized, leading to differences in results from center to center, and making the use of SPT data hard to evaluate in the clinical relevance of the allergenic potential of NFs during RA (Renz et al., 2018).

Previously mentioned *in vitro* and *in vivo* assays have limited diagnostic accuracy since positive results may not be of clinical relevance and are not sufficient to make a definite diagnosis of food allergy. The OFC provides a definitive assessment of a patient’s response to the suspected allergen and is currently considered the most reliable test for diagnosing both IgE-mediated and non-IgE-mediated food allergies (Appel et al., 2018; Santos et al., 2023). Ideally, the OFC should be conducted as a double-blind placebo-controlled food challenge (DBPCFC), with a negative challenge confirmed by a negative open feeding of an appropriate OFC dose. However, DBPCFCs are resource-intensive, requiring the challenge food to be blinded for sensory characteristics such as taste, smell, texture, and appearance. Further considerations need to be made on the preparation of the food (cooked or raw), and the matrix used for blinding, as it could affect the rate of absorption and the dosing regimen (Renz et al., 2018). Moreover, they are time-consuming and patient-unfriendly as they require the ingestion of the potentially allergic food, which can result, in the worst-case scenario, in anaphylactic reactions. The associated risks and ethical concerns make mandatory use of human studies for RA problematic (FAO and WHO Expert Consultation on Allergenicity of Foods Derived from Biotechnology, 2001; FAO and WHO, 2008). Sampson et al. (2024) provide updated, standardized guidelines for conducting an OFC, including pre-challenge assessments, safety precautions, dosing schedules, recommendations on OFC materials, symptom scoring and stopping criteria, as well as guidelines for reporting the results. Such a guideline can be of use when assessing the allergenicity potential of NF that are deemed to require Tier IV testing.

##### 3.3.4.1 Animal studies

In the updated NF guidance (EFSA NDA Panel, 2024), *in vivo* animal studies are not specifically mentioned in the allergenicity section, yet ongoing research indicates these studies might be useful for assessing the allergenic potential of NFs. Although minimizing the amount of animal studies is generally favored to promote animal welfare (EFSA GMO Panel, 2022; FAO and WHO Expert Consultation on Allergenicity of Foods Derived from Biotechnology, 2001; FAO and WHO, 2009), in the future, animal models could be valuable as predictive tools for sensitization and allergenicity assessment, providing a more reliable approach than cellular systems. Various animal study designs focus on allergenicity mechanisms, but their predictive ability for sensitization or elicitation is limited. The clinical relevance of these animal studies as translational models for human food allergy outcomes is still unclear as animals do not entirely reproduce human pathophysiology (Ballegaard et al., 2023; EFSA GMO Panel, 2022; Remington et al., 2018). Several *in vivo* animal models are currently being discussed to target different research aims (Kazemi et al., 2023), among others, Brown Norway rat and BALB/c mice strains models are the most commonly used ones for food allergy because of high IgE responses (Ballegaard et al., 2023; Ballegaard et al., 2019; Dai et al., 2023). However, to date, no animal model has been validated for allergenicity assessment purposes. Challenges such as selecting species and strains which are genetically predisposed to develop an allergy and has human pathophysiology relevance, determining diet, administration routes, doses, test protein formulations, establishing controls and endpoints are still debated to reach a consensus on clinically relevant animal models (Bøgh et al., 2016; Kazemi et al., 2023). In summary, in the future, translating laboratory findings from animal models to human affected by food allergies relevance will require research leading to validated animal models and a deeper understanding of the underlying allergy mechanisms. Validated animal models would be valuable for characterizing both the *de novo* and cross-reactivity immune response to new food allergens in NFs safety assessment.

## 4 Discussion and future perspectives

Allergenicity RA remains a complex and dynamic area within food safety, especially as innovations in food production and biotechnology continue to advance. The growing diversity and complexity of NFs introduces new scientific and regulatory challenges that current allergenicity RA frameworks do not fully address (Fernandez et al., 2024). Currently, allergenicity RA relies primarily on a WoE approach, which often struggles to integrate incomplete or heterogeneous datasets into clear, definitive and actionable conclusions. Although Codex Alimentarius guidelines (Codex Alimentarius Commission, 1985) provide a foundational framework for allergenicity RA, they are primarily designed for individual, purified proteins and do not reflect recent advances in molecular biology, immunology, proteomics, and bioinformatics. While the allergenicity evaluation of single, well-characterized proteins is relatively well established, assessing complex mixtures, such as NFs, presents significant additional challenges (Fernandez et al., 2021; Moreno et al., 2024; Ververis et al., 2020). Recognizing these limitations, EFSA and experts in the field have called for updated methodologies that are better aligned with current scientific knowledge and technologies (EFSA GMO Panel, 2022; Verhoeckx et al., 2020).

**FIGURE 5:**
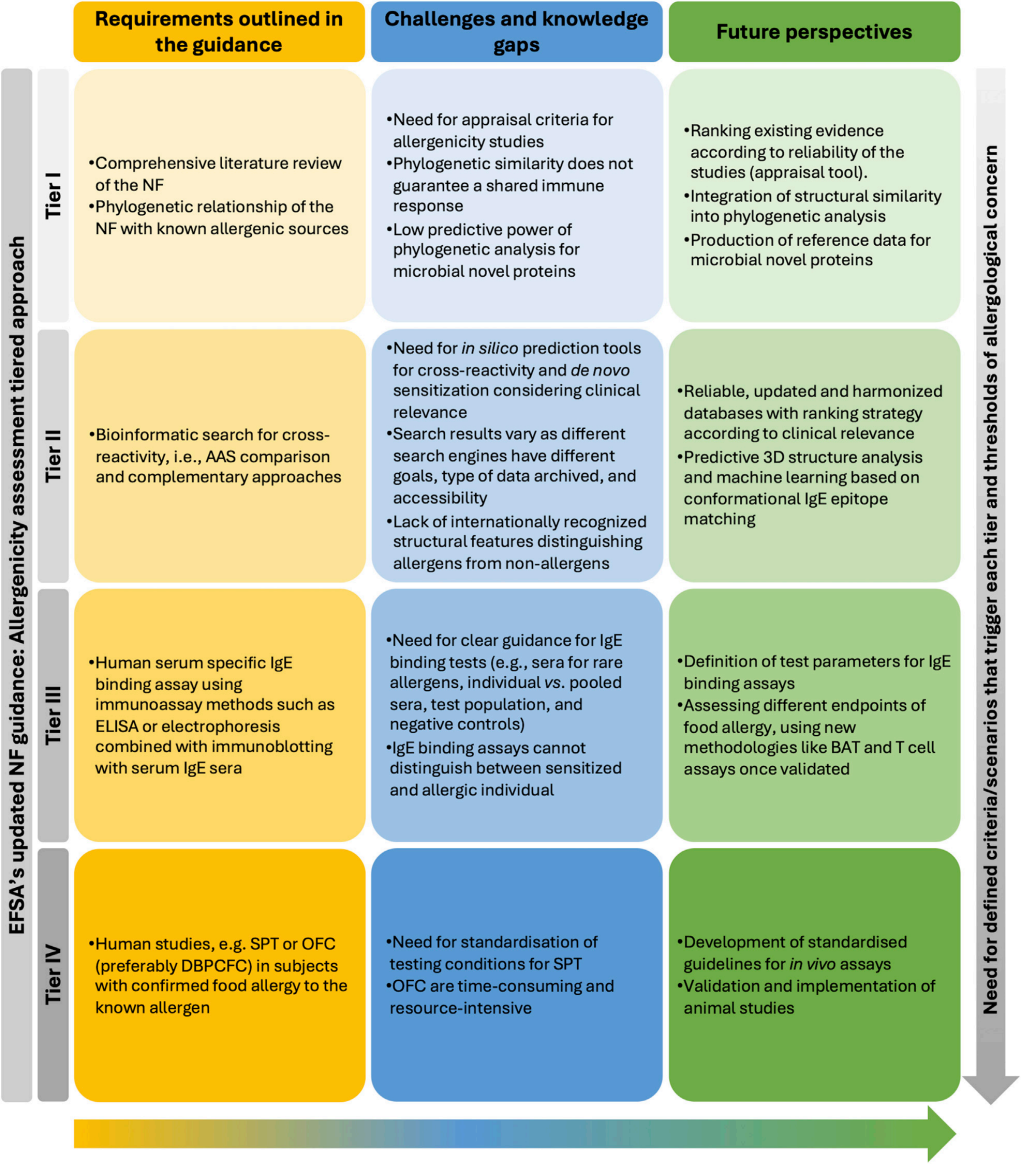
Data requirements in the tiered approach according to the updated EFSA guidance for investigating potential cross-allergenicity of NFs for which their allergenic potential is unknown, challenges and future perspectives in allergenicity RA.

The updated EFSA NF scientific guidance (EFSA NDA Panel, 2024) introduces a more structured and pragmatic tiered approach. However, several aspects beyond the remit of this guidance, require further refinement and the involvement of different stakeholders. Key challenges, knowledge gaps, and future requirements are outlined in Figure 5.

A primary need in allergenicity RA is the implementation of a structured approach for conducting thorough, unbiased literature reviews on the allergenicity potential of the NF or its related sources, using defined appraisal criteria with emphasis on clinical relevance (Mills et al., 2024). By making the most out of the already publicly available knowledge, such an approach could enhance the allergenicity RA of NFs. If sufficiently robust evidence is obtained, this could also reduce the need for additional, potentially unnecessary, testing, thereby decreasing costs and time associated with RA.

Clarification of the role of protein digestibility in allergenicity RA is also essential. Standardized protocols that yield physiologically relevant, reproducible results need to be validated. There is a lack of consensus on interpreting parameters such as resistance to digestion, fragment size post-digestion, peptide aggregation, and allergen abundance. Consequently, the establishment of cut-off values for allergenicity RA purposes is crucial, particularly for complex NFs where digesta characterization is challenging (Verhoeckx et al., 2019). Progress in this area will be key to develop a harmonized allergenicity RA framework based on a better understanding of how various factors influence protein digestibility and, consequently, their immunogenicity and safety.

Further research is needed on phylogenetic relationships to elucidate how the phylogenetic proximity should be interpreted within the prediction of allergenicity potential of a NF (Letovsky et al., 2024). The development of a curated, comprehensive and harmonized database could serve the purpose of advancing in this regard, especially for microorganisms and microbial proteins which are currently poorly investigated (Sarkar et al., 2023).

*In silico* approaches for prediction of cross-reactivity also face limitations, such as database inconsistencies, data accessibility, and annotation levels, which hinder result comparability (Radauer and Breiteneder, 2019; Verhoeckx et al., 2020). To generate reliable and relevant results, robust methods for classifying allergens according to their clinical relevance are needed (Fernandez et al., 2021; Mills et al., 2024). This highlights the need for a centralized and up to date reference allergen database aiming at enhancing consistent and fit for purpose results in RA. Another limitation affecting the reliability of bioinformatic modelling is its primary focus on linear epitopes, while the structural characteristics and 3D morphology of conformational allergenic epitopes remain poorly defined due to the limited availability of experimental data (Huang et al., 2023). Beyond computational tools, *in vitro* (functional) assays can measure cellular responses at different stages of the food allergy reaction (Archila et al., 2016; Santos et al., 2021). While IgE-binding assays are commonly used to evaluate cross-reactivity, these assays cannot differentiate between sensitized and allergic individuals, potentially leading to overly conservative assessments (Remington et al., 2018). On the other hand, emerging *in vitro* assays (e.g., BAT or MAT) are discussed to distinguish between sensitized but tolerant and allergic individuals, offering insights into the clinical relevance of allergic reactions (Santos et al., 2014; Bahri et al., 2018). *In vitro* functional assays, not only offer the advantages to broaden the understanding of cellular immune responses but they are also less invasive, and more cost-effective alternatives to provocation studies. In the long term, ideally, such functional assays may have the potential to reduce time-consuming and resource-intensive *in vivo* testing in the evaluation of potential allergenic risks associated with NFs (Ebo et al., 2024; Santos and Shreffler, 2017). However, their use in RA is currently limited by a lack of standardization, validation and agreement on result interpretation.

While *in vivo* studies such as OFC and SPT are the gold standards for the clinical diagnosis of food allergies, they are very resource demanding and pose ethical challenges (FAO and WHO Expert Consultation on Allergenicity of Foods Derived from Biotechnology, 2001; FAO and WHO, 2009). Therefore, in certain cases, their use for NF RA might be disproportionate. Animal models could represent a useful alternative to support the WoE assessment of NFs, but, besides ethical considerations, they require international consensus, standardized guidelines, and validation before integration into the RA process (Kazemi et al., 2023).

Currently, allergenicity RA of NFs focuses mainly on cross-reactivity. Looking ahead, methods to reliably predict *de novo* sensitization would significantly enhance the RA process of NFs. To date, as immune responses are still insufficiently understood, predictive and validated methods for assessing the risk of *de novo* sensitization caused by novel proteins are still largely lacking, representing a significant gap that urgently needs to be addressed (Verhoeckx et al., 2020). AI could have a critical role in addressing it, as well as in improving the reliability of cross-reactivity predictions in each of the different RA steps considered in the WoE approach (van Breugel et al., 2023). For instance, machine learning could be applied to systematic literature screening, development of clinically relevant appraisal criteria, analysis of protein digestibility parameters, creation of harmonized databases, and AI-driven (conformational) epitope prediction via (three-dimensional) protein modelling.

Overall, agreement on the interpretation of early-tier findings and the establishment of clear decision-making criteria at each stage of tier progression are critical, as they could potentially reduce the need and relevance of other types of tests. To support this, the following points should be regarded as research priorities in allergenicity RA of NFs:

- Development of fit-for-purpose, internationally harmonized databases incorporating information on allergens’ clinical relevance and reference (non) allergenic protein sets;
- Validation of *de novo* sensitization and cross-reactivity assays (both *in silico* and *in vitro*) that meet regulatory standards;
- Improved methodologies to link early experimental and computational findings to clinically relevant allergenic responses;
- Development of a decision-making framework based on standard criteria/thresholds that trigger each tier.

Progress in these areas will depend on the international validation and standardization of new tools and methodologies. Nonetheless, it is crucial to highlight that RA serves the purpose of replying to questions posed to address risk management matters. Thus, the refinement of emerging approaches and the applicability of sometimes resource-intensive testing depends on the scientific and regulatory objectives of allergenicity RA for which there is still need for a shared understanding and consensus. This includes clarity on:

- The overall purpose of the allergenicity assessment;
- The endpoints and parameters that should be evaluated;
- The acceptable levels of uncertainty or confidence;
- The thresholds for defining acceptable versus unacceptable allergenic risk.

Methodological advances in allergenicity RA and the definition of validated methods and threshold criteria could also benefit other decision-making frameworks beyond the RA that are increasingly informing policymaking in the field of food, nutrition and public health (e.g., risk-benefit assessment, multiple criteria decision analysis) (EFSA Scientific Committee, 2024). Additionally, the implementation of post-market monitoring systems, although outside the allergenicity RA framework, could also offer a valuable prospect for future advancements in the assessment prediction of NFs allergenicity. Although challenging, having a consensus on triggers, methods and endpoints for post-market monitoring allergenicity surveillance could improve the predictability of allergenicity potential of certain NFs by linking cause-effect. Moreover, advancing harmonized, validated tools for allergenicity post-market monitoring, could help to detect unexpected allergic reactions in consumers, shedding light on aspects of allergenicity RA that are, to date, difficult to predict. By enabling such connections with decision-making frameworks and tools, allergenicity RA could be evaluated alongside nutritional, environmental, and socio-economic factors, consistent with the One Health paradigm (EFSA Scientific Committee, 2024; Pali-Schöll et al., 2021).

## 5 Conclusion

Addressing the multidisciplinary challenges of allergenicity RA, will require continued collaboration between regulatory bodies, academia, and industry. Harmonized protocols adapted to the realities of modern food innovation are essential. At the same time, continued investment in fundamental research to better understand the mechanisms of food allergy and sensitization is crucial. Through coordinated efforts, transparent data sharing, and a unified research agenda, safety of NF products could be further supported by an updated scientifically robust allergenicity RA framework.

## Footnotes

1 Commission’s priorities. Available here: https://commission.europa.eu/priorities-2024-2029_en

2 European protein strategy. European Parliament resolution of 19 October 2023 European protein strategy (2023/2015(INI)). Available here: https://www.europarl.europa.eu/doceo/document/TA-9-2023-0375_EN.pdf

3 Regulation (EU) 2015/2283 of the European Parliament and of the Council of 25 November 2015 on novel foods, amending Regulation (EU) No 1169/2011 of the European Parliament and of the Council and repealing Regulation (EC) No 258/97 of the European Parliament and of the Council and Commission Regulation (EC) No 1852/2001. Available here: https://eur-lex.europa.eu/eli/reg/2015/2283/oj/eng

4 See Annex A - Public consultation on the draft scientific opinion on the scientific requirements for an application for authorisation of a novel food in the context of Regulation (EU) 2015/2283: https://efsa.onlinelibrary.wiley.com/action/downloadSupplement?doi=10.2903%2Fj.efsa.2024.8961&file=efs28961-sup-0001-Annex+A.pdf

5 Regulation (EU) No 1169/2011 of the European Parliament and of the Council of 25 October 2011 on the provision of food information to consumers, amending Regulations (EC) No 1924/2006 and (EC) No 1925/2006 of the European Parliament and of the Council, and repealing Commission Directive 87/250/EEC, Council Directive 90/496/EEC, Commission Directive 1999/10/EC, Directive 2000/13/EC of the European Parliament and of the Council, Commission Directives 2002/67/EC and 2008/5/EC and Commission Regulation (EC) No 608/2004. Available here: https://eur-lex.europa.eu/eli/reg/2011/1169/oj/eng

6 Available here: https://joint-research-centre.ec.europa.eu/scientific-tools-and-databases-0/toxrtool-toxicological-data-reliability-assessment-tool_en

7 Available here: https://ki.se/en/imm/scirap-science-in-risk-assessment-and-policy/scirap-tools

8 Available here: https://ntp.niehs.nih.gov/sites/default/files/ntp/ohat/pubs/riskofbiastool_508.pdf

9 Available at: https://www.meduniwien.ac.at/allfam/

10 Available at: http://www.allergenonline.org/

11 Available at: https://fermi.utmb.edu/

12 Available at: https://bioinfo.unipune.ac.in/AllerBase/Home.html

13 Available at: https://ddg-pharmfac.net/AllergenFP

14 Available at: https://www.ddg-pharmfac.net/allertop_test/

15 Available at: http://bioinformatics.bmc.uu.se/evaller.html

16 Available at: http://tiger.dbs.nus.edu.sg/AllerHunter

17 Available at: www.imtech.res.in/raghava/algpred/

18 Available at: http://www.allergenonline.org/

19 Available at: https://allergen.org/

20 Last updated in January 2025. Available at: https://comparedatabase.org/

21 Last updated in January 2025. Available at: http://www.allergenonline.org/

22 Last updated in 2021. Available at: https://www.allergome.org/

## Author disclaimer

The author Estefanía Noriega Fernández is currently employed with the European Food Safety Authority (EFSA) in the Executive Director Office - Chief Scientist Office. The authors Andrea Germini, Ermolaos Ververis, Antonio Fernández Dumont, Anna-Maria Pieger (trainee from November 2024 to July 2025) and Marcello Laganaro are employed with the European Food Safety Authority (EFSA) in the Nutrition and Food Innovation Unit that provides scientific and administrative support to the Panels on Nutrition, Novel Foods and Food Allergens and on Genetically Modified Organisms. The author Alejandra Muñoz González is currently employed at the University of Parma and collaborates in the same EFSA Unit as part of the EFSA Guest Scientist Programme (from April 2025 to October 2025). The views expressed in this work are those of the authors and should not be interpreted as representing the official position of EFSA. Thus, the present work is submitted under the sole responsibility of the authors and may not be considered as an EFSA scientific output. EFSA cannot be held accountable for any errors or inaccuracies that may appear.
